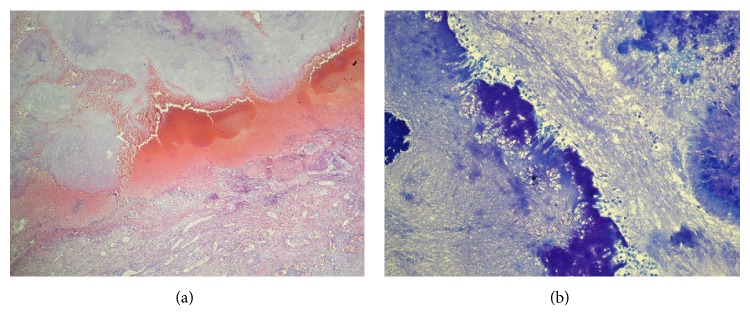# An Unusual Case of Pulmonary Nocardiosis in Immunocompetent Patient

**DOI:** 10.1155/2014/963482

**Published:** 2014-11-17

**Authors:** Zehra Yaşar, Murat Acat, Hilal Onaran, Mehmet Akif Özgül, Neslihan Fener, Fahrettin Talay, Erdoğan Çetinkaya

**Affiliations:** ^1^Department of Chest Diseases, Abant Izzet Baysal University School of Medicine, Gölköy, 14280 Bolu, Turkey; ^2^Department of Chest Diseases, Karabuk University School of Medicine, 78000 Karabuk, Turkey; ^3^Pulmonary Division, Yedikule Chest Diseases and Surgery Teaching and Research Hospital, 34010 Istanbul, Turkey; ^4^Yedikule Chest Diseases and Surgery Teaching and Research Hospital, 34010 Istanbul, Turkey

## Abstract

Pulmonary nocardiosis is a subacute or chronic necrotizing pneumonia caused by aerobic actinomycetes of the genus *Nocardia* and rare in immune-competent patients. A 35-year-old male, who had treated with antituberculosis drugs, presented with cough, dyspnea, and expectoration with episodes of hemoptysis with purulent sputum. The diagnosis of nocardiosis was made by microscopic examination of the surgically resected portion of the lung and revealed filamentous Gram-positive bacteria.

## 1. Introduction

Nocardiosis, caused by Gram-positive, weakly acid-fast, filamentous aerobic actinomycetes, is an opportunistic infection and remains as a possible cause of pulmonary and systemic infection in immunocompromised patients [1]. But it can be isolated in otherwise immune-competent patients that consisted at least 15% of the infections in patients without a definable predisposing condition [2].* Nocardia* species are common natural inhabitants of the soil throughout the world. Pulmonary nocardiosis is usually acquired by direct inhalation of* Nocardia* species form contaminated soil, and person-to-person transmission is rare [3]. Pulmonary nocardiosis is difficult to be diagnosed and is often mistaken for other lung diseases. We report a case of pulmonary nocardiosis that resembled tuberculosis, in a 35-year-old patient without a definable predisposing condition.

## 2. Case Report

**Figure 1:**
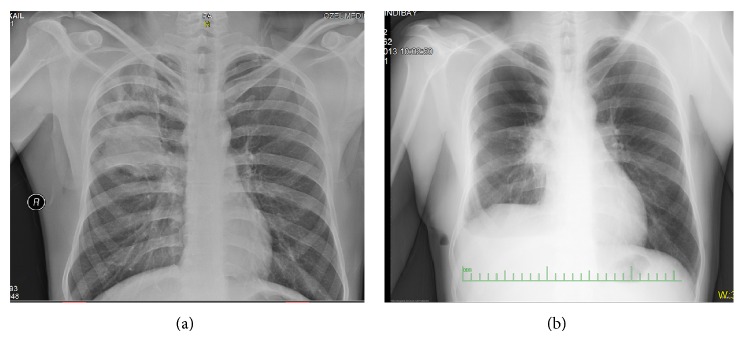


**Figure 2:**
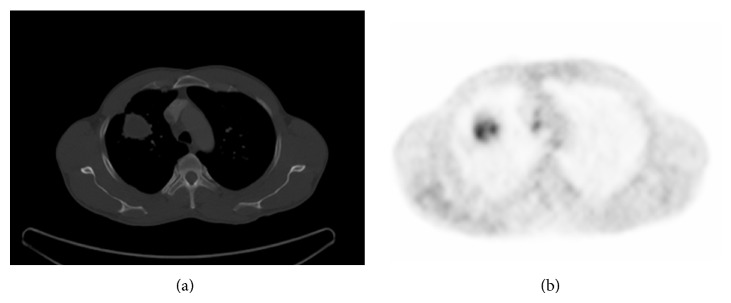


A 35-year-old male presented with cough, dyspnea, and expectoration with episodes of hemoptysis with purulent sputum for 2 years. He took an antitubercular treatment for six months. With antitubercular treatment his fever had subsided but the sputum and hemoptysis had continued. Two months ago he referred to a general physician with low grade fever associated with productive cough and received some medication without any improvement. His condition became worsened. Chest X-ray showed infiltrations in right upper lobe with cavity formation (Figure 1(a)) and computed tomography (CT) revealed the presence of areas of consolidation with air bronchograms and cavitary lesions containing air and infiltration beginning from the apical segment lying to anterior segment of right lower lobe (Figure 2). The FDG PET/BT revealed a hypermetabolic lesion over the right upper lobe of the lung of the patient, with a maximum standardized uptake value (SUV) of 5.9–7.1 which favors a malignancy (Figure 2). Then, he received some antibiotics such as ceftazidime and ciprofloxacin. But he did not have any improvement in respiratory symptoms. Several sputum samples were collected and tested for the presence of acid-fast bacilli, but all smears were negative. The patient then underwent bronchoscopy and aspirated material was negative for tuberculosis, fungi (including Pneumocystis jirovecii), and malignancy. FNAC was done from the right upper lung lesion. Aspirated material was negative for tuberculosis and malignancy. Because of progressive worsening of clinical status, right upper lobectomy was performed. On gram staining, the organism appeared as Gram-positive, thin branching filaments. Modified Ziehl-Neelsen staining showed many branching acid-fast bacilli, consistent with the morphology of* Nocardia* species (Figure 3). The patient was started on trimethoprim-sulfamethoxazole. The patient improved remarkably both clinically and radiographically (Figure 1(b)).

## 3. Discussion

*Nocardia* infection is a rare disorder caused by bacteria, which tends to affect the lung, brain, and skin. Pulmonary nocardiosis is a subacute or chronic pneumonia caused by a species of the family Nocardiaceae. Seven species have been associated with human disease.* N. asteroides* is responsible for about 70% of infection caused by these organisms [4], and debilitated patients have a 45% mortality rate even with appropriate therapy. The typical lesions of nocardiosis are abscesses extensively infiltrated with neutrophils. There is usually extensive necrosis; granulation tissue often surrounds the lesions.

*Nocardia* infections are rare among normal population. Nocardiosis typically develops in immunocompromised persons, such as those suffering from a lymphoreticular malignancy and Cushing's disease, those with acquired immune deficiency syndrome, those with transplanted organs, and those receiving high-dose corticosteroids [5]. Suppression of cellular immunity appears to play a key role in the establishment of* Nocardia* infection [6]. Bronchopulmonary or disseminated nocardiosis can occur in various rheumatologic diseases, including SLE, temporal arteritis, polyarthritis nodosa, intermittent hydrarthrosis, vasculitis, or uveitis [7]. Persons with pulmonary alveolar proteinosis are also at increased risk [8]. Nocardiosis can occur in apparently healthy population but further detailed immunologic evaluation particularly considering interleukin-12-gamma interferon pathway deficiency or other immunologic systems may help in diagnosis of these patients' underlying diseases in the future. Amatya et al. have also reported a case of immunocompetent individual with subcutaneous involvement involving* Nocardia* brasiliensis [9]. In our case any definable predisposing conditions were detected.

The clinical presentation of pulmonary nocardiosis is variable and nonspecific with a chronic course [6]. Symptoms usually have been present for days or weeks at presentation. In this case symptoms were present for two years before referring to our clinic. The usual symptoms are that of dyspnea, productive cough, and fever. In our case presenting symptoms were those of chronic cough with productive sputum, low grade fever, weakness, and failure to respond to ATT (antitubercular therapy).

The chest radiographic manifestations are pleomorphic and nonspecific. Consolidations and large irregular nodules, often cavitary, are most common; nodules, masses, and interstitial patterns also occur [10]. Upper lobes are more commonly involved [3]. Computed tomography findings include consolidation with or without cavitation, multiple discrete pulmonary nodules, pleural effusion, and chest wall extension.

Since the clinical and radiologic manifestations are nonspecific, and the microbiological diagnosis is often difficult, it seems likely that, in some patients, pulmonary nocardiosis will be mistaken for other infections, such as tuberculosis, bacterial pneumonia, or malignancies. In countries where tuberculosis is very common, antituberculous drugs are started on basis of radiology and clinical symptoms like our case. A classic radiographic evidence of tuberculosis that is unresponsive to medication raises the suspicion of other diseases. Kumar et al. reported a case of pulmonary tuberculosis; however in our case the patient was not suffering from pulmonary tuberculosis but was mimicking pulmonary tuberculosis, because of which there was failure to respond to ATT [11]. Similar cases mimicking pulmonary tuberculosis had been reported [12, 13] but invasive diagnostic procedures were not needed for diagnosis like our case.

Difficulty and slowness of culture growth, along with the lack of a serologic test for nocardiosis, necessitate its inclusion in the differential diagnosis for both immunocompromised and immunocompetent patients in whom an apparent pulmonary infection cannot be rapidly diagnosed. If sputum examinations do not yield the diagnosis in a suspected case and the diagnosis cannot be made easily from lesions elsewhere in the body, more invasive diagnostic procedures like bronchoscopy, needle aspiration, and open lung biopsy should be performed [11]. Because of progressive worsening of clinical status and a hypermetabolic lesion over the right upper lobe of the lung which favors a malignancy in our case, open lung biopsy and right upper lobectomy was performed.

The treatment of choice for this infection includes sulphonamides and, more recently, trimethoprim and sulphamethoxazole associated with surgical drainage when required but other regimens like amikacin, imipenem, minocycline, linezolid, and cephalosporins are alternatives [14, 15]. Therapy must be prolonged to prevent relapses. The duration of treatment for nocardiosis depends on disease site. For pulmonary involvement, therapy is usually continued for 6 to 12 months or for 2 to 3 months after disease resolution [16].

This case highlights that pulmonary nocardiosis should be keep in mind in also immunocompetent patients, especially in suspected cases of tuberculosis not responding to antitubercular therapy and showing no tubercle bacilli either in the direct smears or cultures.

## Figures and Tables

**Figure 3:**